# Lung Topology Characteristics in patients with Chronic Obstructive Pulmonary Disease

**DOI:** 10.1038/s41598-018-23424-0

**Published:** 2018-03-28

**Authors:** Francisco Belchi, Mariam Pirashvili, Joy Conway, Michael Bennett, Ratko Djukanovic, Jacek Brodzki

**Affiliations:** 1 Mathematical Sciences, University of Southampton, Southampton, UK; 2 Faculty of Health Sciences, University of Southampton, Southampton, UK; 3 NIHR Southampton Respiratory and Critical Care Biomedical Research Centre. University of Southampton, Southampton, UK; 4 Clinical and Experimental Science, Faculty of Medicine, University of Southampton, Southampton, UK

## Abstract

Quantitative features that can currently be obtained from medical imaging do not provide a complete picture of Chronic Obstructive Pulmonary Disease (COPD). In this paper, we introduce a novel analytical tool based on persistent homology that extracts quantitative features from chest CT scans to describe the geometric structure of the airways inside the lungs. We show that these new radiomic features stratify COPD patients in agreement with the GOLD guidelines for COPD and can distinguish between inspiratory and expiratory scans. These CT measurements are very different to those currently in use and we demonstrate that they convey significant medical information. The results of this study are a proof of concept that topological methods can enhance the standard methodology to create a finer classification of COPD and increase the possibilities of more personalized treatment.

## Introduction

Chronic obstructive pulmonary disease (COPD) is a progressive lung disease, affecting more than 200 million people worldwide. COPD is the fourth leading cause of death in the world and is projected to be the third leading cause of death by 2020. There were more than 3 million deaths from COPD in 2012 worldwide. The global burden on health resources as a result of COPD is expected to rise^1,2^. COPD is characterized by chronic inflammation of the bronchi and the lung parenchyma, resulting in varying degrees of obstructive bronchitis and emphysema due to remodeling of the airways and destruction of the alveoli, respectively. Although its pathology is heterogeneous, in functional terms, all forms of COPD result in loss of lung function, which is usually quantified by measuring the forced expiratory volume in 1 second (FEV1) and the Forced Vital Capacity (FVC). While these spirometry measures are widely used in clinical practice, both to diagnose and stratify COPD by severity, they have important limitations, the main being that they are integrative measurements which, therefore, do not take into account the highly heterogeneous regional pathological changes of COPD^3^. Furthermore, FEV1 correlates weakly with clinical outcomes and health status^4–6^

For the needs of COPD, lung function measurements are increasingly complemented by imaging methods as a means of visually quantifying regional ventilation and perfusion abnormalities, gas trapping, emphysema, and airway remodeling^3^. High-resolution computed tomography (HRCT) scans are the most widely used form of imaging, with MRI and nuclear medicine increasingly, but still less commonly, used. Technical advances have resulted in dramatic reductions in radiation dose of CTs, allowing repeat imaging in longitudinal studies. Assessment of bronchial wall and cross-section thickness is comparable to histological quantification and also enables estimation of the degree of small airways disease that are not directly visualized by CT^7^. Of note, CT imaging allows for detection of lung pathology, such as smoking-related inflammation of the small, distal bronchi (bronchiolitis), years before airflow limitation is detected by spirometry^8^. For example, CT-detected emphysema, assessed by the 15% percentile (Perc15) technique is able prospectively to identify rates of lung function decline, even in individuals in whom spirometry does not detect airway obstruction^9^.

Common CT measurements in COPD research include lung attenuation area, mean lung density, airway wall area percentage, Perc15, lung volume, airway wall thickness and airway lumen area^10^. There is, however, significant room for the development of radiomic features derived by data-characterization algorithms applied to large sets of quantitative features extracted from medical images to uncover characteristics that cannot be appreciated by the naked eye. In the current study, we have applied, to our knowledge for the first time ever, the technique of persistent homology to process lung CT data. We took advantage of the computational tool of persistent homology^11–13^ to create topological descriptors which capture the complexity of the lung structure; this also enabled computation of a measure of similarity between images. Using this approach, our study has introduced a novel set of descriptors computable from a chest CT scan, focusing on characteristics that are very different from those used at present. Specifically, we started by considering three new radiomic features: upwards complexity, which quantifies the way branches stretch upwards, the length of the bronchial tree visible in an inspiratory CT scan, and the number of bifurcations in the same tree. We then showed that these three numerical values are very closely related and any of them can stratify the inspiratory CT scans of our cohort into groups that agree with those given by the GOLD guidelines of COPD. Of note, these stratification results are better than those obtained by other CT measurements, like the emphysema score, volume of the lumen or airway diameter. Apart from the upwards complexity, we also computed two additional numerical values related to the way branches stretch upwards. Using these, we could clearly distinguish between inspiratory and expiratory CT scans. Additionally, we observed that we can also classify our cohort into healthy individuals and COPD patients by quantifying and classifying the topological structure of the space between the lung periphery and the visible airways in an inspiratory CT scan. Finally, we developed a computable characteristic that describes how the branches in the bronchial tree curve towards one another and showed that this radiomic feature correlates with lung function more strongly when the computations are done using the expiratory CT scans rather than the inspiratory CT scans, a phenomenon that is also seen when using standard CT measurements.

We propose that the relation between lung diseases and the shape of the bronchial tree, including properties such as trajectory changes, are of value to advancing our understanding of the mechanisms of COPD. We also propose that further research that applies this method in prospective, longitudinal studies and interventional trials is justified.

## Results

The overall aim of this study was to develop a set of new radiomic features that can distinguish between healthy non-smokers as well as healthy smokers and patients with COPD. For this purpose, the following four study participant groups defined by smoking status and spirometry given by the GOLD guidelines^6^ were studied: healthy non-smokers and healthy smokers (both judged as healthy by spirometry showing FEV1 > 80% of predicted and FEV1/FVC > 0.75), mild COPD patients, consisting of GOLD stage 1 (w**i**th FEV1 ≥ 80% of predicted and FEV1/FVC < 0.70) and moderate COPD patients, consisting of GOLD stage 2 (50% ≤ FEV1 < 80% of predicted and FEV1/FVC < 0.70). See Methods for cohort details and data used.

In this paper, we made use of Topological Data Analysis (TDA), with emphasis on persistent homology, for the computation of our new radiomic features. In Supplementary Information, we explain what persistent homology is and how it works. In Methods, we explain the way we use this TDA tool to obtain each of our geometric signatures.

### Directional complexity

**Figure 1:**
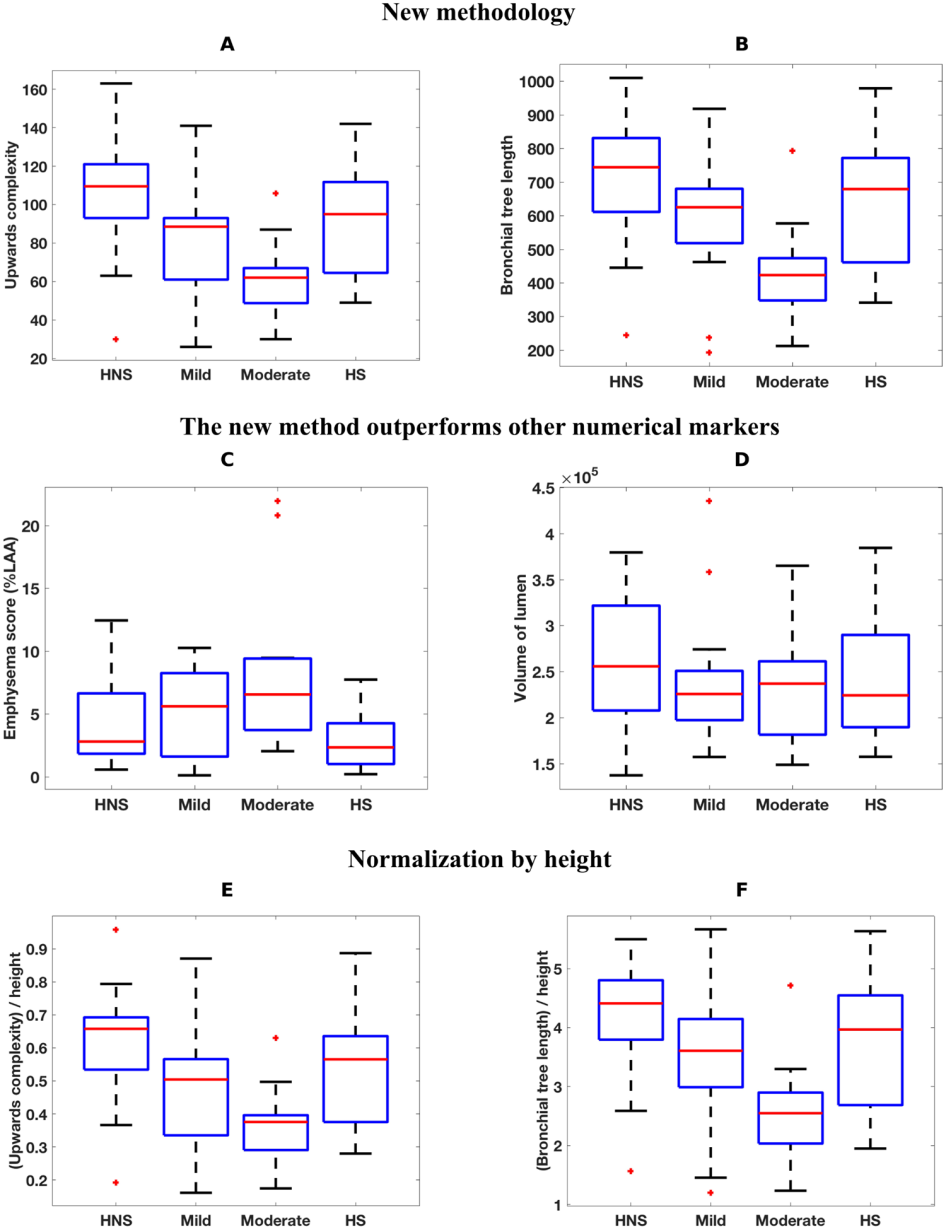
Differences between severity groups given by 6 radiomic features. In the boxplots, HNS = healthy non-smokers, Mild = mild COPD patients, Mod = moderate COPD patients and HS = healthy smokers. The + signs denote outliers. The 6 radiomic features studied are (**A**) upwards complexity (see Methods for details), (**B**) bronchial tree length, (**C**) emphysema score (as percentage of low attenuation area), (**D**) volume of the airways (computed as the number of voxels inside the grey airway structure in Fig. 4A), (**E**) upwards complexity divided by participant’s height, (**F**) bronchial tree length divided by participant’s height. The combination of radiomic features A and B can distinguish all groups except for HS from HNS or from Mild, which outperforms the combination of methods C and D.

**Figure 2:**
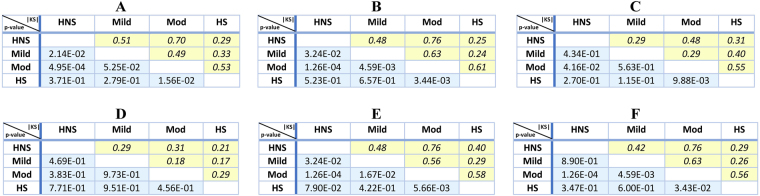
Differences between severity groups given by 6 radiomic features. For each radiomic feature, we show the table with the pairwise Kolmogorov-Smirnov that compares HNS = healthy non-smokers, Mild = mild COPD patients, Mod = moderate COPD patients and HS = healthy smokers. The values in italics in yellow shaded boxes indicate the absolute value of the KS score and the values in roman type in blue shaded boxes indicate p-values. The 6 radiomic features analyzed are (**A**) upwards complexity (see Methods for details), (**B**) bronchial tree length, (**C**) emphysema score (as percentage of low attenuation area), (**D**) volume of the airways (computed as the number of voxels inside the airway structure in Fig. 4A), (**E**) upwards complexity divided by participant’s height, (**F**) bronchial tree length divided by participant’s height. The combination of radiomic features A and B can distinguish all groups except for HS from HNS or from Mild, which outperforms the combination of methods C and D.

For this computation, we began by extracting a graph representing the bronchial tree from each inspiratory CT scan (see Methods). Starting from the top of the scan, we recorded the height at which a segment of the bronchial tree changes direction and starts pointing upwards or downwards. We computed a geometric summary of this information using TDA as described in Methods. This consisted of a single numerical output we call upwards complexity, which was obtained by counting the number of times a particular branch changes its trajectory to start stretching upwards and sum this number over all branches in the bronchial tree. Upwards complexity allowed us to stratify the inspiratory CT scans of our cohort into COPD groups that agree with those given by the GOLD guidelines. A boxplot illustrating this group-separation can be found in Fig. 1A and details of the pairwise Kolmogorov-Smirnov (KS) tests can be found in Fig. 2A. The table in Fig. 2A shows (with the notation introduced there) that upwards complexity was able to distinguish HNS from Mild (|KS| = 0.51, and $p=2.14\times {10}^{-2}$), HNS from Mod (|KS| = 0.7, $p=4.95\times {10}^{-4}$) and HS from Mod (|KS| = 0.53, $p=1.56\times {10}^{2}$).

We also studied how the branches in the bronchial tree bend in other directions, obtaining a different number for each direction. This directional complexity in directions other than upwards did not improve the group-separation results obtained by the combination of upwards complexity and bronchial tree length (introduced in the next section), hence our focus on the latter two measurements. More details on this are given in Methods.

### Length of the bronchial tree and number of branching points

To complement directional complexity, we measured the length of the entire bronchial tree observable in an inspiratory CT scan. The length of the bronchial tree was estimated from a graph representing the bronchial tree in the CT (see Methods) using the number of vertices in this graph as a proxy for the length of the bronchial tree. Using this measure, we could again stratify the inspiratory CT scans of our cohort into groups that agree with those given by the GOLD guidelines. For these group-separation results, see Fig. 1B for the boxplot and Fig. 2B for the results of the pairwise KS test. In particular, notice how the bronchial tree length separates the group of moderate COPD patients from the other three groups.

Moreover, it is remarkable that the length of the bronchial tree was able to distinguish all groups, except for that of healthy smokers (HS) from those of healthy non-smokers (HNS) or mild COPD patients (Mild). To be precise, Fig. 2B shows that the bronchial tree length distinguishes HNS from Mild (with a Kolmogorov-Smirnov score of |KS| = 0.48, and a p-value of $p=3.24\times {10}^{-2}$), HNS from Mod (|KS| = 0.76, $p=1.26\times {10}^{-4}$) and HS from Mod (|KS| = 0.61, $p=3.44\times {10}^{-3}$), as upwards complexity did. In addition, bronchial tree length also separates Mild from Mod (|KS| = 0.63, $p=4.59\times {10}^{-3}$). Of note, these findings may indicate that the group of healthy smokers is heterogeneous and intersects with the healthy non-smokers at one end and with the mild COPD patients at the other. This could not be established using FEV1 (% of predicted) and the ratio FEV1/FVC.

To investigate whether any part of the lung may be contributing more to the above findings, we computed the length of the bronchial tree starting from different airway generations. This showed that such thresholding does not improve the separation presented in Fig. 1B and Fig. 2B regardless of the generation from which the computation began. In a separate computation, an almost identical separation to the one in Fig. 1B and Fig. 2B was reproduced by using the total number of points where airways branch out instead of the length of the bronchial tree.

### Relationship between directional complexity and bronchial tree length

**Figure 3:**
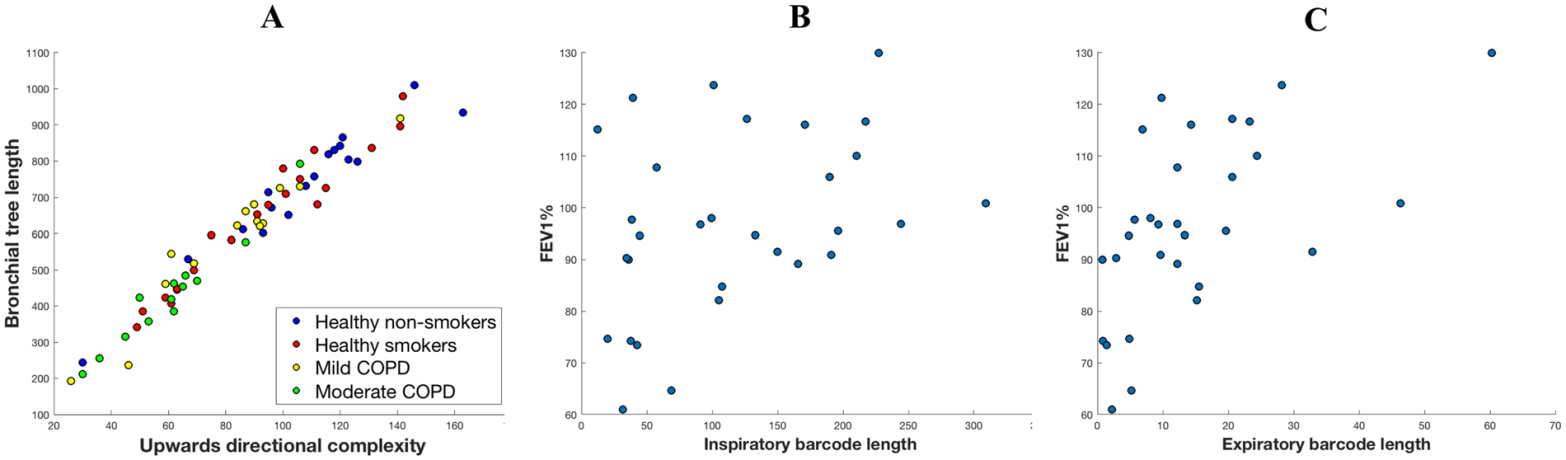
Analysis of topological characteristics. (**A**) Correlation between upwards complexity and bronchial tree length (Pearson correlation coefficient *ρ* = 0.97, p-value *ρ* = 1.56.10^−41^). Similar results were obtained using directional complexity in other directions. (**B**) Correlation between the inspiratory branch-to-branch proximity, which quantifies how branches of the inspiratory bronchial tree bend towards one another, and FEV1 (% of predicted) (*ρ* = 0.38, *ρ* = 0.040). (**C**) Expiratory counterpart of (b) (*ρ* = 0.57, *ρ* = 0.001). Notice that the correlation is stronger and more significant for expiratory scans than for inspiratory scans.

When investigating the directional complexity in any given direction and the length of the bronchial tree, these two measures were found to be strongly related (see Fig. 3A for an illustration of this using upwards complexity). Moreover, as shown in Fig. 3A, this close relation was maintained across the four groups in the cohort. It should be noted that while one can expect directional complexity to be related to the bronchial tree length, the precise nature of this relationship is not at all clear *a priori*.

### Comparison with other analytical methods

**Figure 4:**
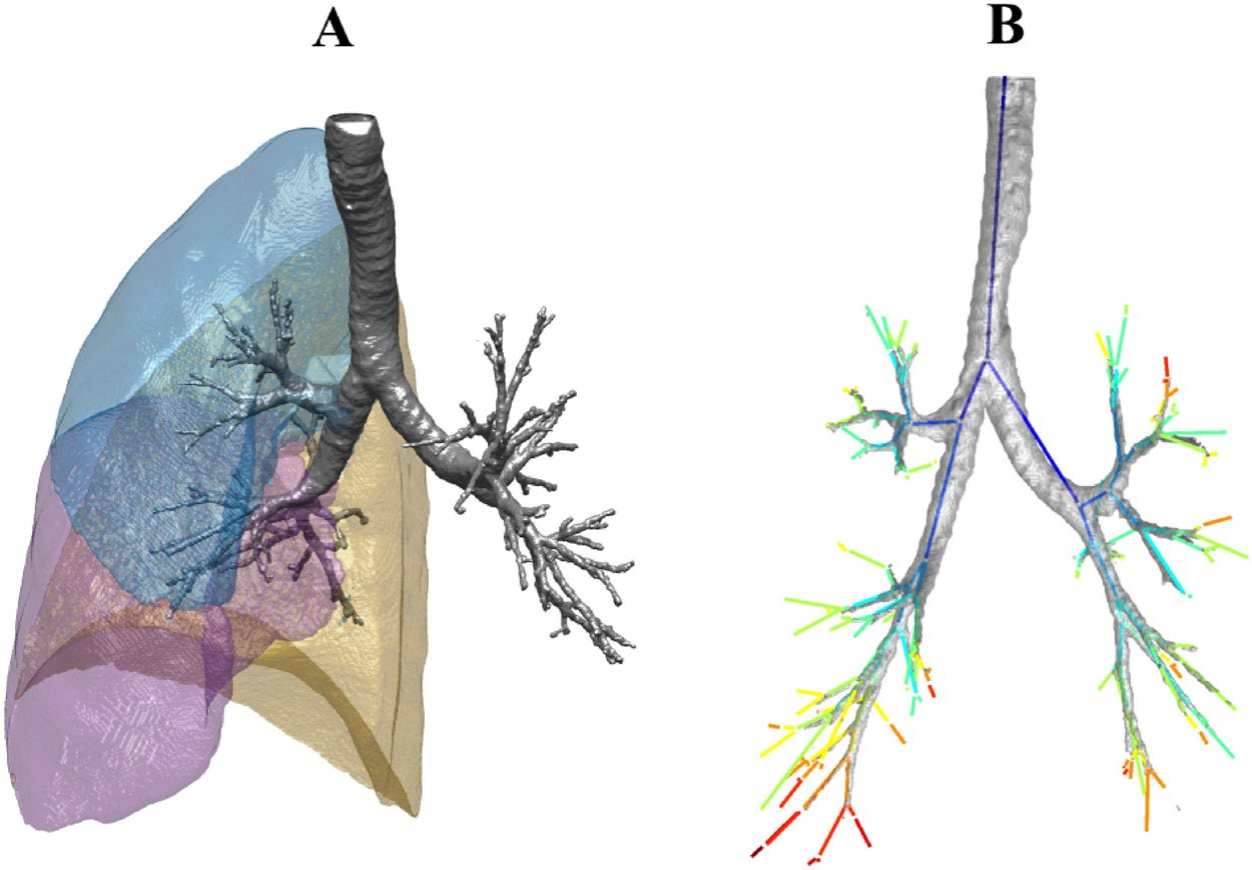
MSCT analysis. (**A**) The Apollo software (Vida Diagnostics, Iowa, USA) was used to segment the lobes from the MSCT scans. The resulting contour of the right lung lobes is presented here using custom software written in Matlab (R2015b, MathWorks, Natick, MA 01760–2098, US). (**B**) Illustration of the extracted branch center lines, along with the segmented airway tree for one of the participants. The center lines are colored according to generation number. Note that for the purposes of illustration, the center lines are plotted between the branch points only. For all of the analysis described in this paper, the complete center line information was used, which captured the true shape of the airways as in Fig. 8.

Having observed significant separation between study groups using our new radiomic methods on inspiratory CT scans, we looked for similar differences between subject groups when using other CT measurements. Specifically, we quantified emphysema using the standard measure of percentage area of low attenuation and we approximated the volume of the airway lumen as the number of voxels inside the airways (see the grey airway structure in Fig. 4A). This showed that the differences between subject groups identified by our radiomic features were much more significant than the differences identified by the emphysema score and the volume of the lumen (compare the boxplots Fig. 1C and Fig. 1D with those in Fig. 1A and Fig. 1B, and the numerical results in Fig. 2C and Fig. 2D with those in Fig. 2A and Fig. 2B). Indeed, using the volume of the lumen, we did not find any difference between subject groups with a p-value < 0.05, and the emphysema score only found two such differences – namely, that between healthy smokers and moderate COPD patients ($|{\rm{KS}}|$ = 0.55, $p=9.88\times {10}^{-3}$) and that between healthy non-smokers and moderate COPD patients ($|{\rm{KS}}|$=0.48, $p=4.16\times {10}^{-2}$). These separations were weaker and less significant than the separation of the same groups obtained using the bronchial tree length (Fig. 1B and Fig. 2B).

### Relation to height

When assessing standard lung function measurements, the values are typically normalized by the individual’s height. To study the effect of height on some of our new radiomic features, we divided the upwards complexity of each participant by the person’s height. We then examined the effect of normalization by height on group separation. To this end, we compared 2-sample Kolmogorov–Smirnov tests. Normalizing upwards complexity exhibited a clearer separation in 3 cases (using the notation in Fig. 1, those cases are the comparisons Mod-HNS, HS-Mod and Mod-Mild) and a less clear separation in 2 cases (Mild-HNS, HS-Mild) (compare Fig. 1E and Fig. 2E to Fig. 1A and Fig. 2A, respectively).

We repeated the same normalization with the length of the bronchial tree and found that in 2 instances (HNS-Mild, HS-Mod), not normalizing by height provided a clearer separation between groups, and normalizing did not improve the clarity of separation in any instance (compare Fig. 1F and Fig. 2F to Fig. 1B and Fig. 2B, respectively). This suggests that, unlike standard spirometry, the bronchial tree length may capture bronchial structure information relevant in COPD in a way that is independent of height.

### Comparison of expiratory and inspiratory phase CTs

For 30 participants (8 healthy non-smokers, 9 healthy smokers, 8 mild COPD and 5 moderate COPD), both inspiratory and expiratory CT scans were obtained. This provided an opportunity to demonstrate first that our methodology can not only distinguish between healthy individuals and COPD patients but also detects differences in structure between the inspiratory and expiratory phases of the breathing cycle. Furthermore, we showed that the amount to which branches in the bronchial tree curve towards one another correlates with lung function more strongly in expiratory CT scans than in inspiratory ones. The stronger correlation with expiratory scans is in keeping with our previous findings using standard CT measurements that mean lung density (MLD) during expiration correlated better with reduced lung function than inspiratory MLD^14^.

**Figure 5:**
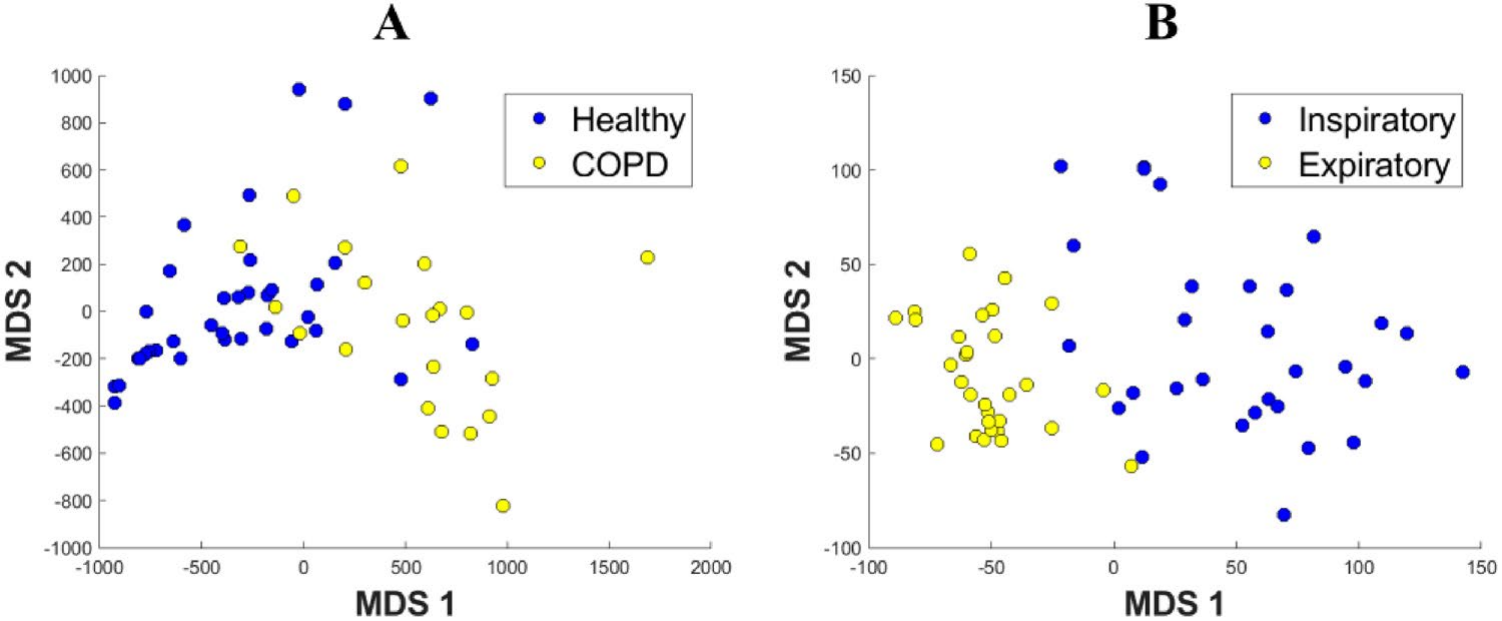
Spatial representation of similarities between lungs. These are obtained by describing the shape of each lung through a set of topological characteristics called barcodes (see Methods for details) and computing distances between the barcodes of individual subjects. The resulting space is represented in 2D through an MDS embedding. In the legends, Healthy = healthy smokers and non-smokers, COPD = mild and moderate COPD patients. (**A**) This representation uses degree-2 persistent homology of inspiratory data to infer the shape of the airways inside the cavity of the lobes and it shows a clear distinction between Healthy and COPD groups. The overlap between the groups suggests that our characteristics are on a continuous spectrum. The presence of two nominally healthy cases so deep in the COPD region suggests a potential undiagnosed problem. Interestingly enough, those two individuals were healthy smokers. Similarly, all 6 COPD points which lie in or just outside the healthy region correspond to mild COPD patients. (**B**) This representation takes into account how the airways bend upwards and shows that this topological feature clearly separates the inspiratory and expiratory stages of the bronchial tree. This analysis was not performed for the expiratory phase because the information about the lobe structure was not available.

To address the first point, we considered again the height at which branches in the bronchial tree start or stop stretching upwards, as used in the computation of upwards complexity. We used the same input to compute a different topological summary (see Methods), which allowed us to compare the scans of different participants and plot them together. The output of our computations were two values per CT scan, which we used as coordinates of a point in the plane (see Fig. 5B). This showed a clear separation between the inspiratory and expiratory CT scans.

**Figure 6:**
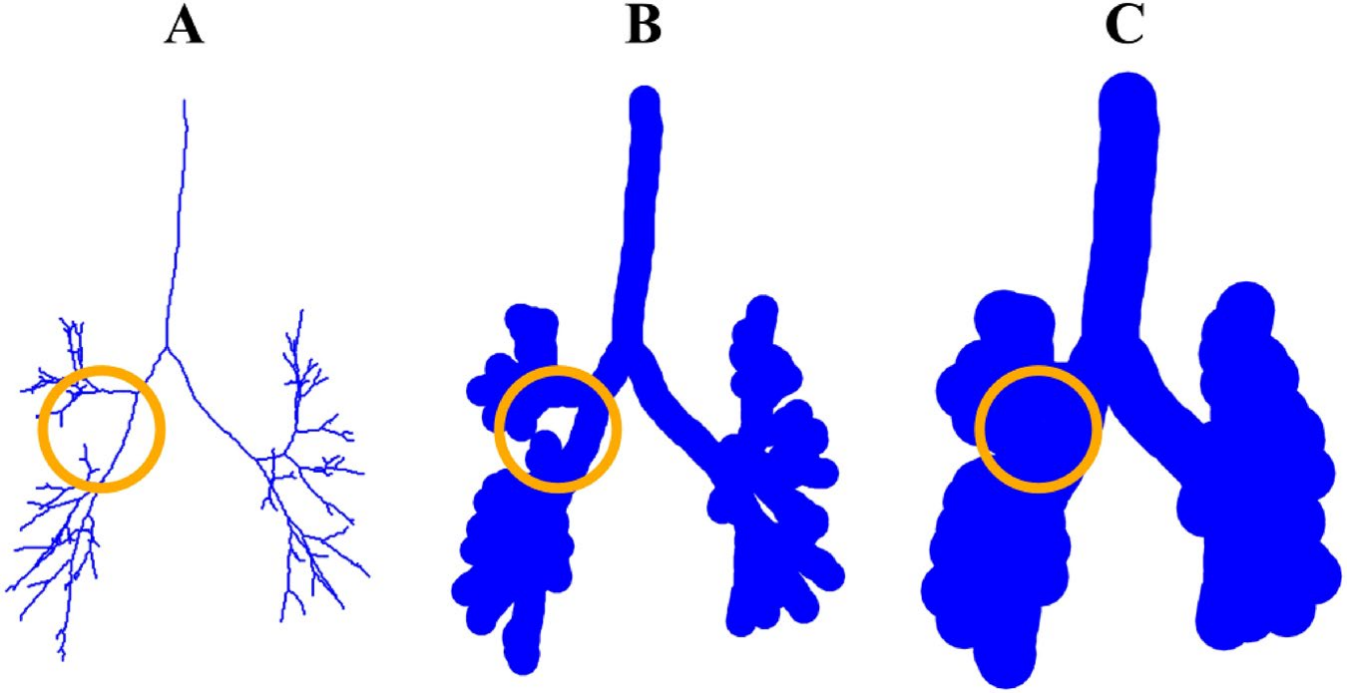
Computing of branch-to-branch proximity. Consider the graph representing the bronchial tree as explained in Methods (**A**). This graph is called a tree since it contains no loops, i.e., no branches that bifurcate and then merge. Of note, there are many nodes (up to 264) between any two consecutive bifurcations, so the nodes appear dense in the graph representation. Centered at each node of this graph, we virtually set a ball of a fixed radius, thickening the construction. As we keep thickening more and more, by increasing the radius of those balls, at some point we will find that some branches merge, creating a loop (**B**). We record the radius *r*_1_ at which this happens. For a large enough radius *r*_2_, though, this loop will be filled in (**C**). If a merging of branches creates a loop that appears for the value *r*_1_ of the radius and disappears at *r*_2_, we represent this merging as the positive number *r*_2_ − *r*_1_. Summing up all these terms, we obtain a number we call branch-to-branch proximity.

**Figure 7:**
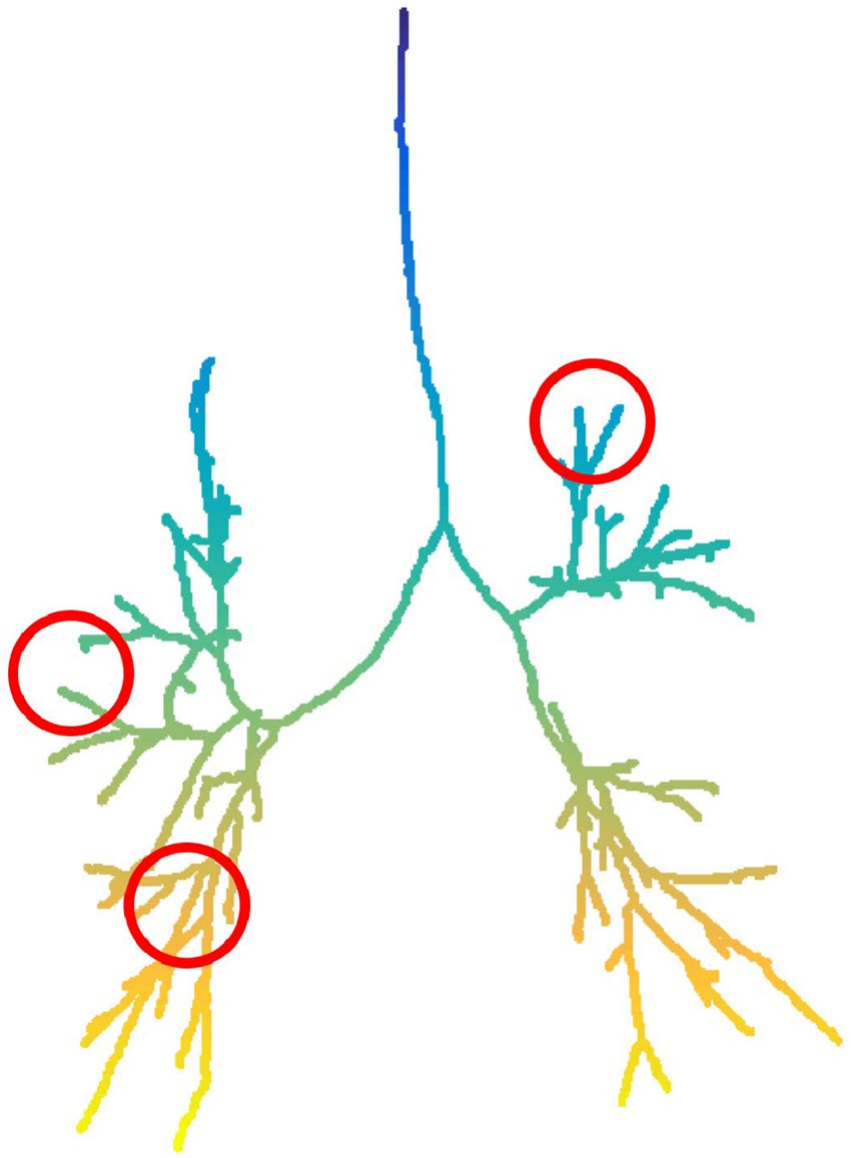
Calculations show that the lung function is better when more branches bend towards one another in the expiratory bronchial tree (such as the branches in the two circles on the left, in contrast with those in the circle on the right). See Fig. 3C.

To quantify how branches curve towards one another in the bronchial tree graph, we introduced another radiomic feature, called *branch-to-branch proximity*. This was done by virtually thickening the visible airways and recording the thickness at which the airways begin to touch (see Fig. 6 and Fig. 7 for an illustration). Using this approach, we found that the branch-to-branch proximity observed in the expiratory phase correlated more strongly with FEV1 (% of predicted) than the branch-to-branch proximity observed in the inspiratory phase (compare Fig. 3B and Fig. 3C). Again, this was consistent with standard CT measurements, which also correlate better with FEV1 (% of predicted) when measured during expiration^10,14^.

### Small airways

Having shown that we can compute clinically meaningful topological features of the airways by using their tree structure, we showed that the shape of the space separating the lung periphery from the airways visible in an inspiratory CT scan is also related to the development of COPD. Our CT scan data consists of voxels which are cubes 0.7 mm long in each direction, giving a spatial resolution of 2.1 mm. This makes the small airways, which are defined as those with a diameter <2 mm, invisible in a CT scan. However, it is well known that small airways dysfunction plays a key role in COPD. Hence, what happens in the void between the visible airways and the lung periphery is crucial. To overcome the relatively low resolution of the standard CT scan, we provided numerical topological characteristics of the shape of the space between the airways and the boundary of the lobes. We demonstrated how the resulting radiomic feature can distinguish between healthy individuals and COPD patients. This was achieved by placing virtual balls centered within the visible structure (airways and lung periphery) and allowing them to expand until they fully occupied the space. This procedure uses thickening in a similar way to Fig. 6, see Methods for details. The output is a pair of real numbers (*x, y*) representing each CT scan. In Fig. 5A, we represent each CT scan as a point in the plane with the corresponding coordinates (*x, y*). This approach placed the healthy smoking and non-smoking individuals into one group that was distinct from the mild and moderate COPD patients who formed another group (see Fig. 5A).

## Discussion

Since its creation, persistent homology, a key tool of TDA, has developed rapidly, both in terms of its mathematical foundations^15–17^ and possible applications. Persistent homology has been used in fields as diverse as digital imaging^18,19^, sensor networks coverage^20^, materials science^21,22^, molecular modelling^23–25^, signal processing^26,27^ and virus evolution^28^. In this study, we took advantage of the ability of persistent homology to offer alternative ways of measuring global properties of complex objects through the use of topology.

In respiratory imaging, this method represents a completely new way of taking measurements of the bronchial tree. In contrast to existing methods, such as measuring the dimensions of the airway walls and lumen, which look at airway branches individually, our approach condenses the topological properties of the entire bronchial tree into a small number of unique characteristics for each individual. Along with previous studies^29,30^, this creates major new opportunities for persistent homology to be used more widely in medicine, in particular in clinical radiology. This new methodology is especially applicable to studying the lung at the population level because of the manner in which it represents the complexity of the airway tree through a single low-dimensional data point that represents the entire bronchial tree. By collecting these data over a large number of subjects and combining them with other imaging, physiological and measurements of pathobiological biomarkers, we could build a picture of how variations in the topological nature of the bronchial tree impact on the pathophysiology of people with a variety of respiratory diseases such as COPD, asthma and idiopathic pulmonary fibrosis (IPF). This is likely to have significant translational impact as a valuable tool for use in deep-phenotyping, which is central in stratified medicine and precision medicine^31^.

We have created a set of novel radiomic features which capture the overall complexity of the lung structure and enable a quantitative comparison of the CT scan images. We have demonstrated that these properties are important in the context of a common respiratory disease. These measurements provided a more complete picture of differences between the four groups in this study than standard CT measurements. In particular, there was a significant relationship between upwards complexity, the length of the bronchial tree, and COPD severity. Moreover, it is remarkable that the length of the bronchial tree was able to distinguish all groups, except for that of healthy smokers from those of healthy non-smokers or mild COPD patients, which may indicate that the group of healthy smokers is heterogeneous and intersects with the healthy non-smokers at one end and with the mild COPD patients at the other. This could not be established using FEV1 (% of predicted) and the ratio FEV1/FVC.

Additionally, our comparison between inspiratory and expiratory phases can have various applications. The manner in which tissue inside the lung expands and contracts throughout the breathing cycle is known to be an indicator of disease, for example gas trapping in COPD. Comparison of the inspiratory and expiratory scans can therefore be exploited as a means of making localized measurements of disease^32^. Our proposed technique makes it possible to study how the shape of the bronchial tree changes during the breathing cycle and offers the potential to be a new method for the identification of localized areas of disease, such as gas trapping. Similarly, indices that are the subject of current, clinical, pulmonary CT research also include the Parametric Response Mapping (PRM) technique, which uses co-registration of paired inspiratory and expiratory scans to compare areas of low attenuation on a voxel to voxel basis^33,34^. Our methodology may further inform the PRM technique and could be the subject of future research.

The precise physiological basis for our results is not completely clear at this stage. Upwards complexity counts the number of times a branch deflects upwards from the horizontal direction. Lower directional complexity means that there are fewer airways pointing upwards from a given plane. The same applies to downwards complexity, or indeed complexity evaluated in any direction in three dimensions. Our interpretation is that advancing smoking disease (fall in FEV1) is associated with increased distortion of the bronchial tree structure, which results in some of the airways pointing in a direction that is different from the other neighbouring airways, possibly because of differences in air trapping. Thus, regional differences in the extent of lung damage (emphysema) could explain distortions that result in individual airways departing from the direction of the other neighbouring airways that have different degrees of air trapping. A natural pathophysiological explanation for the association between FEV1 and branch proximity is that it reflects the distortion (squashing) of the airways by hyperinflated lung parenchyma, also due to air trapping. As can be seen in Fig. 2C, this squashing is more pronounced in expiration, as shown by the stronger and more significant correlation in this respiratory phase (see *p* and *p* values, respectively).

Our approach to persistent homology is similar to that employed in^30^ to study cerebral vasculature, but our topological summaries, such as the directional complexity or branch-to-branch proximity, are different and have not been used before. As explained in Supplementary Information, the output from the persistent homology calculation is summarized in the so-called barcode. In the study of cerebral vasculature, Bendich *et al*.^30^ simplified the analysis by retaining the 100 longest bars from which summaries were produced using the Principal Component Analysis. In contrast, in our study this approach did not work as the number of bars can vary significantly between patients, which can be seen in Fig. 1A where some participants have about 160 bars in the upwards complexity barcode, whereas others exhibit only about 20 bars. For this reason, in our study we retained the entire barcode without thresholding. From this input data, we computed a measure of similarity between scans of individual patients. This can be used for visualization or to train classification models in a way similar to the approach by Adcock *et al*. on hepatic lesions^29^.

We also created a new topological characteristic to circumvent the relatively low spatial resolution of CT scans. This is one of the main mathematical novelties of the paper as it provides a first instance where topology has been used to infer the structure of the object under study. We achieved this by incorporating the boundary of the lung lobes into the computation. This technique can be applied to any kind of imaging, for example, to 3-dimensional Magnetic Resonance Angiography images of the arterial tree within the brain^30^, where the new characteristics developed here can be used to enhance the analysis if the meninges are used in the same way we used the outer layer of the lobes in our study.

Of note, we made use of persistent homology in degrees 0, 1 and 2 in different ways to obtain different kinds of clinical insight. In degree 0, it was used to define the directional complexity (a number that can distinguish severity groups) and to characterize the distinction between the inspiratory and expiratory CT scans. Using persistent homology in degree 1, we showed that CT measurements correlate with FEV1 (% of predicted) more strongly during the expiratory phase than in the inspiratory phase, which could be expected based on similar observations in past CT studies^10,14^. As stated before, the degree 2 was used to overcome the limitation of the low spatial resolution of CT scans by including information of the outer boundary of the lobes. This led to a much clearer visualization of the difference between the healthy and COPD participants, which could not be recovered using the topological characteristics in degrees 0 and 1. Thus, our methodology provides one of the first significant uses of the second-degree persistent homology in applications. The other uses of degree 2 up to date are summarized in^19,35,36^.

In summary, this study has shown that our analytical method can extract information from CT scans to provide a new perspective on lung structure. Because this method can be readily applied to large CT datasets, we propose that it is of value for clinical research. Further studies are needed to assess its prognostic value in longitudinal and interventional studies.

## Methods

All the procedures explained in the Results section can be formalized and computed efficiently through the tool of persistent homology, which is described in detail in the Supplementary Information.

### Study design and participants

The imaging data used for this study were acquired from two previous imaging studies performed in Southampton (manuscripts in preparation). Both studies focused on COPD and had identical inclusion and exclusion criteria. In both studies, participants were recruited into two COPD groups; GOLD stage 1 disease (FEV1/FVC ratio <0.70 and FEV1 ≥80% of predicted) and GOLD stage 2 disease (FEV1/FVC ratio <70% and FEV1 50–79% of predicted), referred to as mild and moderate COPD, respectively. Both healthy smoker and healthy non-smoker groups had no clinical evidence of obstructive airways disease, and had spirometry results of FEV1/FVC ratio >0.75 and FEV1 >80% of predicted. In total, 64 participants were assessed (18 healthy non-smokers, 19 healthy smokers, 14 COPD GOLD-1 and 13 COPD GOLD-2). Both studies were approved by the Southampton and West Hampshire local research ethics committee (LREC number: 11/SC/0319 and 09/H0502/91). Written informed consent was obtained from all study participants and both studies were conducted according to all relevant guidelines and regulations.

### MSCT imaging

All CT scans were performed using a standardized protocol recommended for use with Apollo analysis software (Vida Diagnostics, Iowa, USA). Multi-Slice Computed Tomography (MSCT) scans were performed on a Siemens Sensation 64 CT scanner (Siemens Medical Solutions, Erlangen, Germany) using a high-resolution algorithm, with detector thickness 0.75 mm, pitch 1.0, effective mAs 90 and a tube voltage of 120 kV. The high-resolution algorithm was chosen to ensure the best visualization of the airway tree^37^. The scanning protocol was performed at maximal inspiration and expiration, with breath hold, for the duration of the scan (approximately 10–15 sec depending on the size of the thorax). All patients are coached beforehand and during the scan in a standardized manner, conforming with the Vida Diagnostics protocol and as used by other centers^38,39^. The images were reconstructed using a slice thickness of 0.75 mm, a reconstruction increment of 0.5 mm, and a sharp reconstruction algorithm. Additional reconstructions were also performed using several soft reconstruction kernels, including B30f and B35f, which were chosen to suit the recommended protocol in the Apollo analysis software.

### MSCT analysis

The Apollo software (Vida Diagnostics, Iowa, USA) was used to perform the analysis of the multi-slice computed tomography scans. This software was designed to semi-automatically analyze pulmonary MSCT imaging data, including segmentation of the lungs, the airway tree and the lobes (see Fig. 4A). For the needs of the current study, only the lung, lobes and airway tree were of interest. In many cases, the Apollo software was able to achieve the desired results entirely automatically, but for some participants it was necessary to manually edit the results of the lobe segmentation to ensure that they were defined as accurately as possible. All segmentations were manually inspected for accuracy and completeness. In the case of the lobes, accuracy was checked by ensuring that the lobar boundary corresponded as closely as possible with the fissure, whilst for the airways the segmentation was inspected to ensure that the results were as complete as possible.

Custom software written in Matlab (R2015b, MathWorks, Natick, MA 01760–2098, US) was used to extract the specific details of the center lines and branch points of the airway tree from the data output by the Apollo software. An example of the extracted center lines, along with the segmented airway tree is shown in Fig. 4B, where the center lines are colored according to generation number and are, for simplicity, plotted between the branch points only. For all of the analysis in this paper, the complete center line information was used, which captured the true shape of the airway branches. In particular, there can be up to 264 extra points describing the shape of the bronchial tree between two branch points.

### Persistent homology

Persistent homology^11–13^ has been designed to provide numerical information about the key features of an object under study at a range of scales, which can be regarded as variable resolution at which the object is viewed. A starting point of this process is a simplified approximation of the object, which grows as the scale parameter *r* is varied. In this study, we made use of two different approximations: one based on alpha complexes (see Supplementary Information) and one based on a notion of height function, explained in the directional complexity section (below) and expanded in the Supplementary Information. We computed topological characteristics of the chosen approximation, using all scales at once. These are numerical invariants obtained by computing homology groups *H*_*n*_ of the approximation, and tracing the life-span of features as they appear and disappear with the changing scale. For each degree $n\ge 0$, this information is represented in the form of a collection of intervals with multiplicities, called the degree-*n* barcode explained in detail in the Supplementary Information. These intervals have the form (*r*_1_,*r*_2_) for different values of the changing parameter *r*, and are also known as *bars*, hence the name barcode for a collection of these. Intuitively, degree-0 gives information about the evolution of the connected components along the sequence of growing representations of the object under study. Similarly, degree 1 indicates the evolution of the loops or holes, and degree 2 captures the evolution of cavities or voids, etc. We compared the resulting barcodes using pseudo-distance functions, the Wasserstein and Bottleneck pseudo-distances being examples with important stability properties; see Supplementary Information.

In summary, we used persistent homology to take a growing approximation of an object, compute the associated degree-n barcode for some $n\ge 0$, and compare the corresponding barcodes of different objects using the Bottleneck or Wasserstein distances.

### Directional complexity

**Figure 8:**
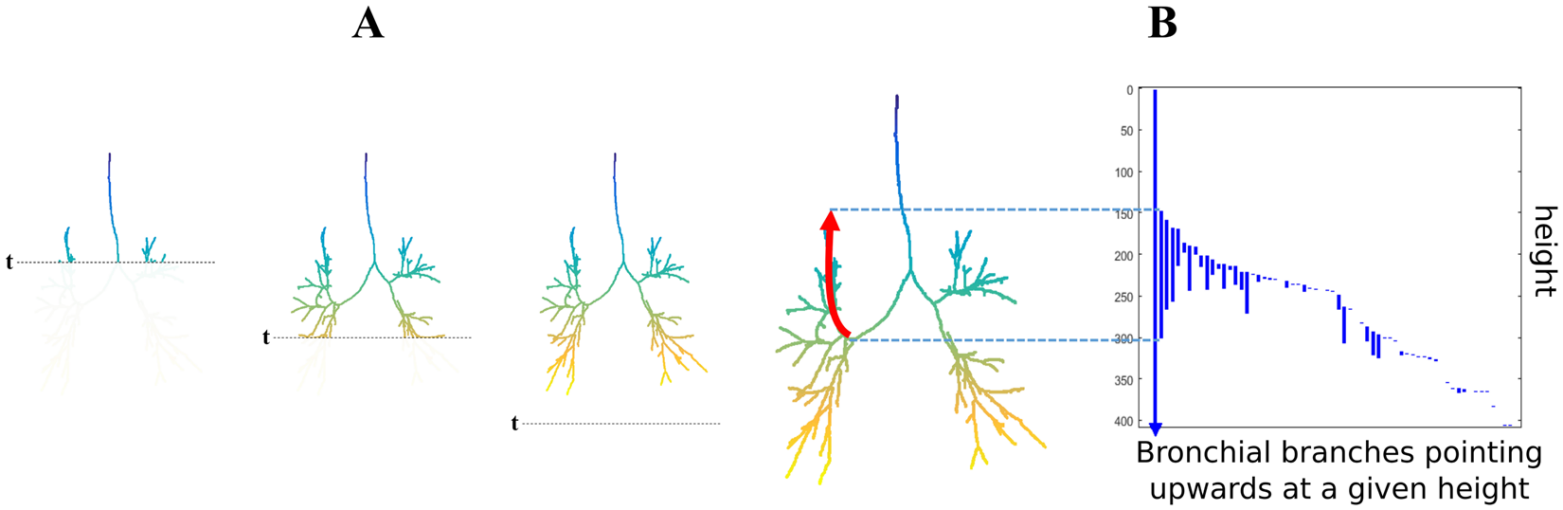
Explanation of upwards complexity. The color gradient indicates height. (**A**) To study upwards complexity, we slide a horizontal plane downwards. If we denote by *X*_*h*_ the part of the tree that sits above the horizontal plane at distance *h* from the top of the image, then ${{X}}_{{h}}\subseteq {{X}}_{{h}\text{'}}$ whenever ${h}\le {{h}}^{^{\prime} }$, obtaining a sequence of nested graphs approximating the bronchial tree more accurately as we increase *h*. (**B**) The right part of the panel shows the degree-0 barcode of the sequence of nested graphs in (**A**). In this picture, the correspondence between bars in the barcode and branches that change trajectory upwards becomes apparent. In particular, the length of a bar indicates for how long a branch follows that upwards trajectory.

We quantified the amount of changes in trajectory in a particular direction by defining a notion of directional complexity on the 3D graph representation of the bronchial tree described in the MSCT analysis subsection (above). To measure upwards complexity, we slid a horizontal plane downwards (see example in Fig. 8A). At any given distance *h* from the top of the imaginary box containing the bronchial tree, *X*_*h*_ was defined as the part of the tree that sits above the plane at that position. In this way, we obtained an approximation of the bronchial tree that converged to the original tree as we increased the distance h from the top (see Fig. 8A).

The degree-0 barcode corresponding to this sequence of growing graphs has the following interpretation: a bar of the form (*h*_1_, *h*_2_) in this barcode indicates that there is a connected component *C* in the graph ${X}_{{h}_{1}}$ which is not present in *X*_*h*_ for any $h < {h}_{1}$. Additionally, the following holds for ${h}_{2}$ but it does not hold for any $h < {h}_{2}$: in the graph ${X}_{{h}_{2}}$, the component represented by *C* will merge with another component of ${X}_{{h}_{2}}$ which was present in ${X}_{h}$ for some $h < {h}_{1}$.

In Fig. 8B, we represent each bar of the form (*h*_1_, *h*_2_) in the degree-0 barcode as a vertical line, with the starting point at distance *h*_1_ from the top and end point at distance *h*_2_ from the top. In this representation, every bar corresponds to a branch changing trajectory to start stretching upwards. We called upwards complexity the number of vertical lines in such a representation, *i.e*., the number of upwards changes of trajectory of the airways.

To compute directional complexity in other directions, we rotated the bronchial tree, slid the plane top to bottom and counted the number of finite bars in the corresponding degree-0 barcode.

As mentioned in the Results section, directional complexity in other directions did not improve the group-separation results obtained by the combination of upwards complexity and bronchial tree length, hence our focusing on the upwards direction. For instance, by rotating 10°, 220° and 0° around the *X*, *Y* and *Z* axes, respectively, following the right-hand rule, directional complexity produced no group separation at all. However, using instead the angles 20°, 40° and 0°, respectively, the group-separation results given by directional complexity were very similar to those of the bronchial tree length.

To generate these barcodes, we used the publicly available software package TDATools^40^. To compute the barcode of one of these graphs in a 3D box, we used the function ‘rca1mfscm’ of this package, which requires the definition of a function $F$ on each vertex and edge of the graph. For instance, to compute the upwards complexity, we assigned to each vertex its distance to the top of the box, and to each edge, the maximum of the values of *F* attained at the two vertices it connects. Note that all barcodes in this study were computed with coefficients in the field of two elements, ${{\mathbb{Z}}}_{2}$.

### Length of the bronchial tree

The length of the bronchial tree was estimated from the 3D graph representation of the bronchial tree described in the MSCT analysis subsection above. We used the number of vertices in this graph (that include not only the branch points but also the many vertices connecting consecutive branch points) as a proxy for the length of the bronchial tree. As stated in the Results section, a separate computation with only the branch points was performed and the results were similar to those in Fig. 1B and Fig. 2B.

### Small airways

For the computation of the representation in Fig. 5A, we started with a 3D array of binary voxels representing the luminal surface of the airways together with the surface of the lobes as in Fig. 4A. For each binary voxel image, we constructed a point cloud in ${{\mathbb{R}}}^{3}$ by including the coordinates of every voxel with value 1 and then built the alpha complex filtration (see Supplementary Information) on these points. The degree-2 barcode of this filtration gave information about how the airways fill the cavity of the lobes. The alpha complex filtrations and their barcodes were computed using the GUDHI library^41^.

Next, we computed the bottleneck distances between all the degree-2 barcodes. This gave a measure of distance between the lung scans by proxy, giving us a pseudo-metric on the set of lungs. The bottleneck distances were computed using the Hera software^42^. Due to computational constraints, we made use of the software’s approximate bottleneck calculation. If one supplies a relative error, then the software computes an approximate distance which satisfies the inequality

$$|{d}_{exact}-{d}_{approx}|/{d}_{exact} < relative\,error,$$

where ${d}_{exact}$ is the exact bottleneck distance and ${d}_{approx}$ is the computed approximation, as described in the documentation of 42. We used a relative error of 10^−4^. After measuring the pairwise distances between all barcodes, we used Multi-Dimensional Scaling (MDS) to obtain a 2D representation shown in Fig. 5A.

### Expiratory CT analysis

For 30 participants (8 healthy non-smokers, 9 healthy smokers, 8 mild COPD and 5 moderate COPD), both inspiratory and expiratory CT scans were obtained. Recall that the upwards complexity was computed as the number of vertical lines in Fig. 8B. This was, in turn, the number of bars in the degree-0 barcode constructed by considering the part of the bronchial tree graph that sits on top of a horizontal plane that we slide downwards.

In order to compute the representation shown in Fig. 5B, we used the same degree-0 barcode in a different way. We computed such barcodes for both the inspiratory and expiratory bronchial tree graphs and compared those 60 barcodes (corresponding to the inspiratory and the expiratory bronchial tree of the 30 patients) using the Wasserstein2 distance (see Supplementary Information). The Wasserstein computations were done with the software package Hera 42. After calculating the distances between all these barcodes, the final representation in Fig. 5B was obtained using a 2D MDS projection.

In a separate computation, we quantified how branches bend towards one another on the tree graph (See Fig. 6 and Fig. 7 for an intuitive illustration of this computation). We used the alpha complex filtration (see Supplementary Information) built on the nodes of this graph. Next, we computed the degree-1 barcode, which consisted of points of the form $({r}_{1},{r}_{2})$. Finally, we defined the branch-to-branch proximity as the sum of the numbers ${r}_{2}-{r}_{1}$ corresponding to all such points. We performed this for both the inspiratory and expiratory tree graphs of each participant. The alpha complex filtrations and their barcodes were computed using the GUDHI library^41^.

### Data and materials availability

The data that support the findings of this study are available on request from the corresponding author (J. B.). The data are not publicly available due to them containing information that could compromise research participant privacy.

## Electronic supplementary material

Supplementary Information
