# Supplementary material for: Lung Topology Characteristics in patients with Chronic Obstructive Pulmonary Disease
Source: Sci Rep. 2018 Mar 28;8:5341. doi: 10.1038/s41598-018-23424-0 (PMC5871819; doi:10.1038/s41598-018-23424-0)
Supplement: Supplementary file 1 — Supplementary Information [file 41598_2018_23424_MOESM1_ESM.pdf]

Lung Topology Characteristics in patients with Chronic Obstructive Pulmonary Disease

Francisco Belchi[1]

Mariam Pirashvili[1]

Joy Conway[2,4]

Michael Bennett[3,4]

Ratko Djukanovic[3,4]

Jacek Brodzki[1,5]

Supplementary Information

[1] Mathematical Sciences, University of Southampton, UK
[2] Faculty of Health Sciences, University of Southampton, UK
[3] Clinical and Experimental Science, Faculty of Medicine, University of Southampton, UK
[4] NIHR Southampton Respiratory and Critical Care Biomedical Research Centre
[5] Correspondence should be addressed to J. B. (J.Brodzki@soton.ac.uk)

Supplementary Information:

Supplementary Methods

Persistent homology

The main mathematical method used in this paper to analyze the lung CT scans is called persistent homology[11,12,13]. It has been designed as a computational way to capture the shape of objects depending on the scale at which they are viewed. To understand the basic idea of persistence, imagine a given set of high resolution images of a human face. If one zooms in, one can capture tiny details of the face, but one may not be able to recognize the person in the photo. Zooming out, one sees less detail, but it will be easier to see the person in the photograph. Continuing the process of zooming out, eventually all details are lost. It is clear that the choice of zooming scale depends on the kind of information we are hoping to recover. We can avoid making a particular choice of zooming scale, and instead study all possible scales at once. This is the approach of persistent homology to study data – it presents us with information about the shape of our data at a range of scales, controlled by a parameter r. For small values of r, we see single points; as r increases, connections between points begin to emerge, creating an approximate shape of the data.

The data set to be analyzed is typically thought to be a discrete subset S sampled from a metric space. To understand the structure of the set, and so capture the information it contains, one creates approximations of S by simple shapes K_r, called simplicial complexes, for a range of values of the scale parameter r. We define the notion of simplicial complex in Section 3 below. As r increases, the corresponding complexes will grow and their structure will also change. Persistent homology will exhibit the evolution of these approximations. Intuitively, homology in degree zero describes the components of the set, in degree one it uncovers the existence of non-trivial loops at a particular scale, while degree two identifies voids or cavities.

This topological information is represented in the form of a set of intervals or bars with multiplicities, called the barcode (see Figure 9). The long bars, which represent features that persist over a wide range of values of the scale parameter, represent significant features of the underlying space the data was sampled from, while short bars typically (but not always) represent noise. Two barcodes can be compared by computing their distance, which provides a measure of similarity. Thanks to the stability theorem, this comparison is robust with respect to noise and small-scale perturbations. We now give details of this process.

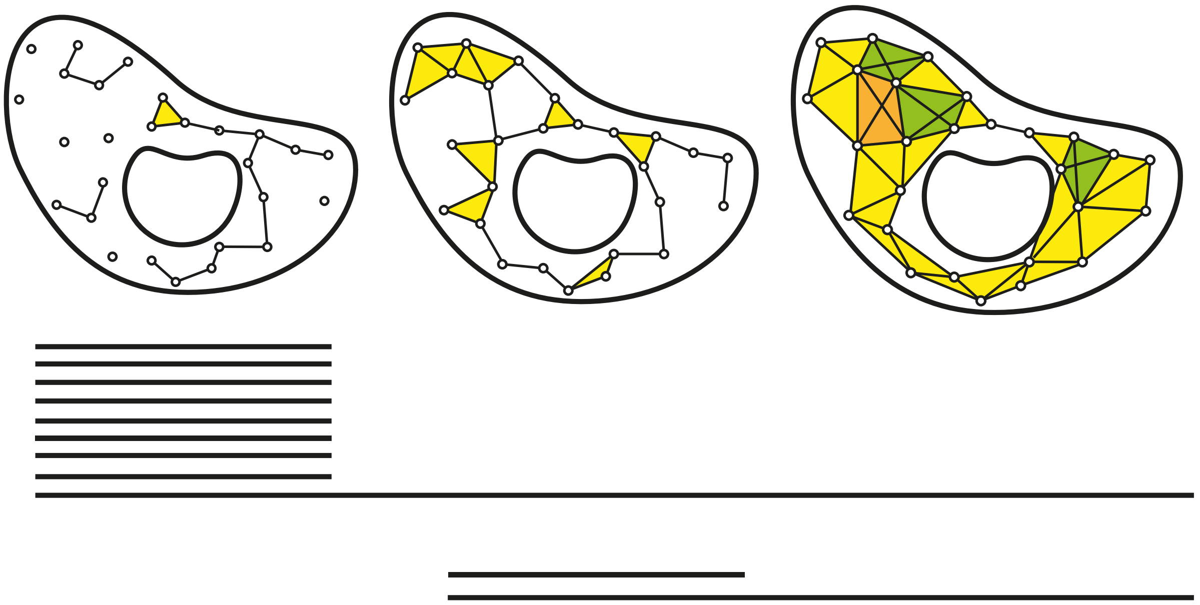

Figure 9 A point cloud is sampled from a deformed annulus in the plane. The sequence of pictures from left to right shows simplicial representations of the set at different scales. In the picture on the left, there are nine components, represented by the horizontal bars below the picture. As the scale increases, all the components combine into one as we move from the left to the middle picture, and this persists for all remaining scales. There are no loops in the left picture, but two loops emerge at the middle scale, represented by the two bars at the bottom. Increasing the scale parameter from the middle to the right picture causes one of those loops to disappear, while the other one persists. Thus, the topological signal is that we have a 'shape consisting of one piece with a hole in it'.

Persistence modules and barcodes

A starting point of persistent homology is the notion of a persistence module V, which is a family of vector spaces over some field $\mathbb{F}$ and linear maps of the form:

$$V_0 \xrightarrow{f_0} V_1 \xrightarrow{f_1} \cdots \xrightarrow{f_{n-1}} V_n,$$

in which composing the consecutive maps starting from some V_i to V_j we get linear maps $f_{i,j}: V_i \to V_j$, for any $0 \le i \le j \le n$. In particular, $f_{i,i}$ is the identity map and $f_{i,i+1} = f_i$. A particularly simple persistence module, denoted by $I_{[i,j-1]}$, which plays a special role in the theory, is obtained as follows. Fix i and j, such that $0 \le i \le j-1 \le n$ and consider the following persistence module

$$0 \to \cdots \to 0 \to \mathbb{F} \xrightarrow{id} \cdots \xrightarrow{id} \mathbb{F} \to 0 \to \cdots \to 0$$

where $\mathbb{F}$ is the ground field considered as a 1-dimensional vector space over $\mathbb{F}$. The first and the last nontrivial terms appear at the places i and $j-1$ respectively. More complex examples are obtained by taking sums of these simple modules in the following sense. If V and V' are persistence modules, then their direct sum $V \oplus V'$ is the persistence module V'', where

$$V_i'' = V_i \oplus V_i', \quad \text{and} \quad f_i'' = \begin{pmatrix} f_i & 0 \\ 0 & f_i' \end{pmatrix}.$$

A theorem by Gabriel[43] states that any persistence module is a direct sum of persistence modules of the form $I_{[i,j]}$. Hence, a persistence module V can be fully characterized by a finite set $D(V)$, called the persistence diagram, which contains a point $(i,j) \in \mathbb{R}^2$ $(0 \le i \le j < n)$ for every summand of

the form $I_{[i,j-1]}$ appearing in the decomposition of V (and a point of the form $(i, \infty) \in \mathbb{R} \times (\mathbb{R} \cup \{\infty\})$ for every summand of the form $I_{[i,n]}$). Each point in $D(V)$ appears with multiplicity equal to the number of copies of the corresponding summand. For technical reasons, all points in the diagonal $\{(x, y) \in \mathbb{R}^2 \mid y = x\}$ are added to $D(V)$ as well.

A barcode is a graphical representation of V equivalent to the persistence diagram $D(V)$. It is a collection of intervals with multiplicities[44]. The barcode of V consists of one interval (or *bar*) of the form $[i, j)$ for every off-diagonal point (i, j) in $D(V)$, which describes the range of values of the scale parameter over which a particular feature persists. The multiplicity of an interval is that of its corresponding point in $D(V)$. Figure 9 shows an example of a barcode.

Simplicial complexes

The persistence modules most commonly used in topological data analysis arise from filtered simplicial complexes, whose combinatorial nature is very suitable for computations.

A simplicial complex K with vertex set S is a family of nonempty, finite subsets of S. Subsets of S of $p + 1$ elements are called p-simplices. A p-simplex is represented as a list of its vertices $[v_0, \dots, v_p]$. In a simplicial complex K, one requires that all elements v of S form 0-simplices $[v]$ in K, and if $\sigma \in K$ and $\emptyset \neq \tau \subset \sigma$, then $\tau \in K$. We usually consider the case when S is finite. A simplicial complex K has the associated space $|K|$, called the geometric realization, which can be regarded as a triangulated polyhedron in an appropriate Euclidean space. The combinatorial structure of K can be used to define the so-called p^{th} homology $H_p(K)$ of $|K|$ for all $p \geq 0$. To define $H_p(K)$, we first define the space of p-chains $C_p(K)$ to be the vector space consisting of all finite sums of the form $\sum_\sigma a_\sigma \sigma$, where σ runs through all p-simplices, and a_σ is an element of a ground field $\mathbb{F}$. Typically, coefficients a_σ are taken from a finite field $\mathbb{Z}_\text{p}$ of integers modulo p, for instance $\mathbb{Z}_2 = \{0,1\}$, which was also used in our computations. These vector spaces are connected by the boundary homomorphism $\partial: C_p(K) \to C_{p-1}(K)$. This map is defined on p-simplices $\sigma = [v_0, \dots, v_p]$ by

$$\partial(\sigma) = \sum_k (-1)^k [v_0, \dots, \widehat{v_k}, \dots, v_p],$$

and then extended by linearity. Here the symbol $\widehat{v_k}$ means that the corresponding element v_k is omitted. The next step is to define a vector space $Z_p(K)$ of p-cycles of K, which consists of all vectors $v \in C_p(K)$ such that $\partial(v) = 0$, and a vector space $B_p(K)$ of p-boundaries of K, which consists of all $v \in C_p(K)$ such that $z = \partial(w)$, for some $w \in C_{p+1}(K)$. It is important to note that $\partial \circ \partial = 0$, that is, performing this operation twice sends every simplex to zero. This guarantees that $B_p(K)$ forms a vector subspace of $Z_p(K)$. Hence, it makes sense to define the quotient space, which is called the pth homology of K:

$$H_p(K) = {Z_p(K)}\big/{B_p(K)}.$$

The dimension of $H_p\ (K)$ is known as the pth *Betti number* β_p, of $|K|$. Intuitively, β_0 computes the number of connected components of the geometric realization of K. Likewise, β_1 computes the number of 1-dimensional holes, β_2 computes the number of 2-dimensional holes, etc.

If L is also a simplicial complex on the set of vertices T, such that T is a subset of S and any simplex σ of L is also a simplex of K, then L is called a subcomplex of K and we write $L \subset K$. It follows that $C_p(L) \subset C_p(K)$. If z is a p-cycle in L, it is also a p-cycle of K and if z is a p-boundary in L, it is also

a p-boundary in K. Hence, there is a well-defined map $f: H_p(L) \to H_p(K)$, $p \geq 0$, which is called the induced map. In general, the induced map is not injective, even though $Z_p(L) \subset Z_p(K)$.

A filtered complex K is a nested sequence of subcomplexes

$$K_0 \subset K_1 \subset \cdots \subset K_n.$$

Choosing a homology degree $p \geq 0$, we can write all homology groups and induced maps as a sequence

$$H_p(K_0) \xrightarrow{f_0} H_p(K_1) \xrightarrow{f_1} \cdots \xrightarrow{f_{n-1}} H_p(K_n)$$

that forms a persistence module. The *degree-p barcode* of $|K|$ is defined as the barcode of this persistence module.

Comparing persistence diagrams

There is a number of ways to compare persistence diagrams [45]. If X and Y are persistence diagrams, then the bottleneck distance between X and Y is defined by

$$d_B(X,Y) = \inf_{\gamma} \sup_{x} \|x - \gamma(x)\|_\infty$$

where γ runs through all bijections from $X \to Y$, while x runs through all points of X and for a point of the form $z = (a,b) \in \mathbb{R} \times (\mathbb{R} \cup \{\infty\})$, one has $\|z\|_\infty = max\{|a|,|b|\}$, and $\|(a,\infty) - (b,\infty)\|_\infty = |a-b|$. The qth Wasserstein distance $(q \geq 1)$ is defined by

$$d_{W_q}(X,Y) = \inf_{\gamma} \left(\sum_{x} \|x - \gamma(x)\|_\infty^q \right)^{\frac{1}{q}}.$$

These expressions define pseudo-metrics, as it is possible to create distinct persistence diagrams for which either of these distances is zero.

Stability

The stability theorem for persistent homology, due to Cohen-Steiner, Edelsbrunner and Harer[46], is easier to state in terms of tame functions on triangulable spaces, that is, on spaces which can be represented as a simplicial complex.

Let X be a triangulable topological space and let $f: X \to \mathbb{R}$ be a real-valued function on X. A homological critical value of f is a real number a, for which there exists an integer p such that for all sufficiently small $\varepsilon > 0$ the map $H_p(f^{-1}(-\infty, a-\epsilon]) \to H_p(f^{-1}(-\infty, a+\epsilon])$ induced by inclusion is not an isomorphism. So, the number a corresponds to the value at which the homology of sub-level sets changes. A function f is tame if it has a finite number of homological critical values and the homology groups $H_p(f^{-1}(-\infty, a-\epsilon])$ are finite dimensional for all $p \geq 0$ and $a \in \mathbb{R}$. Typical examples of such functions are Morse functions on compact manifolds and piece-wise linear functions on finite simplicial complexes. For a real number r, one sets $V_r = H_p(f^{-1}(-\infty, r])$. If $r < t$, we have $f^{-1}(-\infty, r] \subset f^{-1}(-\infty, t]$ and therefore we can consider the induced linear map $V_r \to V_s$, which is an isomorphism, if the interval $[r, s]$ contains no homological critical value of f. Hence, varying r, one obtains a finite number of distinct vector spaces V_{r_i}, leading to a persistence module

$$V_{r_0} \to V_{r_1} \to \cdots \to V_{r_n}.$$

In particular, we have a corresponding persistence diagram $D(f)$. The classical stability theorem reads as follows[46]:

Theorem 1. *Let X be a triangulable topological space with continuous tame functions $f, g: X \to \mathbb{R}$. Then the persistence diagrams satisfy*

$$d_B\big(D(f), D(g)\big) \leq \|f - g\|_\infty.$$

In other words, persistence diagrams are stable under possibly irregular perturbations of the function used to create the diagram. In our particular case, this theorem ensures that imprecisions of measurement, such as small differences in the alignment of lungs when the scans were taken, will not lead to a drastic change in the resulting barcodes. There are similar results in terms of Wasserstein distances [46].

Height filtration

In data analysis, a given data set can typically be approximated in several different ways by a family of simplicial complexes. In choosing a suitable representation, one is guided by the properties of the set and computational efficiency. Such a representation is fixed by choosing a real-valued tame function f and computing its sublevel sets $f^{-1}(-\infty, t]$ as in the section on Stability above.

For instance, given a 3D object X, a commonly used function $f: X \to \mathbb{R}$ is that which sends each point $(x, y, z) \in X$ to its "height", i.e. its third coordinate, z. In the paper, to compute the upwards complexity of the graph representation X of a bronchial tree, we use a function very similar to this: for each vertex v in X, we define $f(v)$ as the vertical distance from v to the highest point in the CT scan, and for each edge e in X connecting two vertices v and v', we define $f(e) = max\{f(v), f(v')\}$.

For functions like these, the bars in the degree-0 barcode have a clear interpretation as changes in trajectory. In the case of upwards complexity, those are airway trajectories that change to face upwards.

Alpha complexes

Another construction we use are 3D alpha complexes, which can substantially reduce the computational complexity. To describe this construction, first let us say a word about *Voronoi diagrams*. Given a set S of points in Euclidean space $\mathbb{R}^n$, one defines convex polytopes V_s, $s \in S$ called Voronoi cells, which consist of all points $x \in \mathbb{R}^n$ such that $dist(x, s) \leq dist(x, s')$ for any other $s' \in S$. The subsets V_s give a tessellation of $\mathbb{R}^n$.

Given a finite set of points $S \subset \mathbb{R}^n$ and a real number $r \in \mathbb{R}^n$, one defines the region $R_s(r) = \bar{B}_s(r) \cap V_s$, where $\bar{B}_s(r)$ is the closure of the ball of radius r centered at s. Now we can form the α-complex (or alpha complex) K_r as follows: a subset $\sigma \subset S$ is called an α-simplex if

$$\bigcap_{s \in \sigma} R_s(\sigma) \neq \emptyset.$$

See Figure 10 for an illustration of this construction.

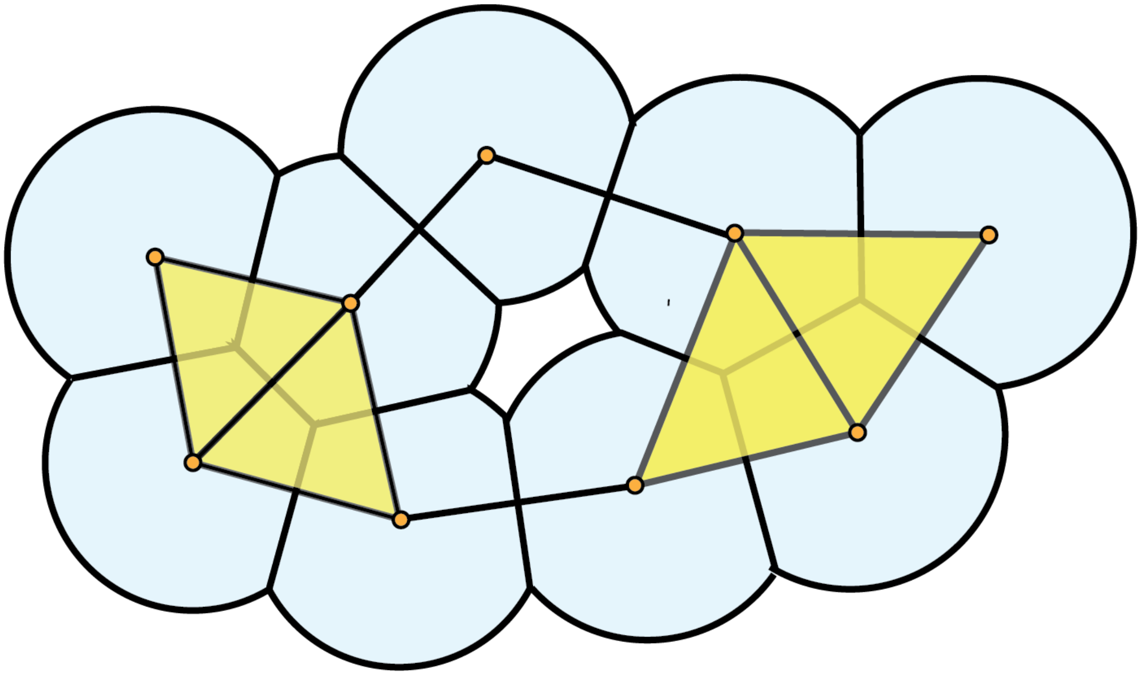

Figure 10 An example of a system of Voronoi cells constructed for a particular value of the scale parameter on a subset of the plane (shown in blue). Superimposed is the alpha complex that represents the structure the set at this scale. The topological signal here is that, at this scale, the points were sampled from a deformed annulus.

Varying r, one obtains a finite family of nested α-complexes

$$K_{r_0} \subset K_{r_1} \subset \cdots \subset K_{r_n}.$$

This is a typical example of a filtered complex. Hence, one can apply the machinery of persistent homology. In particular, we have the corresponding persistence diagrams. This geometric construction can also be phrased in terms of tame functions as before, and thus fits into the same general framework.

In the Methods section in the paper, we applied the alpha complex filtration to two sets of points in $\mathbb{R}^3$. On the one hand, we used the vertices of the 3D graph representation of the bronchial tree described in the Methods subsection called MSCT analysis. The degree-1 barcode of the alpha complex filtration on this collection of vertices provided additional information about the complexity of the branching structure of the airways. On the other hand, we also used a 3D array of binary voxels representing the luminal surface of the airways together with the surface of the lobes as in Figure 4A of the paper. For each binary voxel image, we constructed the point cloud in $\mathbb{R}^3$ by including the coordinates of every voxel with value 1. The degree-2 barcode of the alpha complex filtration on this set of points gave information about how the airways fill the cavity of the lobes.

References

43. Gabriel, P. & Unzerlegbare Darstellungen, I. Manuscr. Math. 6, 71–103 (1972).

44. Ghrist, R. Barcodes: the persistent topology of data. Bull. Amer. Math. Soc. (N.S.) 45, 61–75 (2008).

45. Kerber, M., Morozov, D. & Nigmetov, A. Geometry Helps to Compare Persistence Diagrams, presented at 2016 Proceedings of the Eighteenth 7 Workshop on Algorithm Engineering and Experiments (ALENEX), (unpublished) (2016).

46. Cohen-Steiner, D., Edelsbrunner, H. & Harer, J., Stability of Persistence Diagrams (Symposium on computational geometry, 2005).
